# Cardiovascular Effects of Cannabidiol: From Molecular Mechanisms to Clinical Implementation

**DOI:** 10.3390/ijms26199610

**Published:** 2025-10-01

**Authors:** Hrvoje Urlić, Marko Kumrić, Nikola Pavlović, Goran Dujić, Željko Dujić, Joško Božić

**Affiliations:** 1 Department of Cardiology, University Hospital of Split, 21000 Split, Croatia; hurlic@kbsplit.hr; 2 Department of Pathophysiology, University of Split School of Medicine, 21000 Split, Croatia; marko.kumric@mefst.hr (M.K.); nikola.pavlovic@mefst.hr (N.P.); 3 Laboratory for Cardiometabolic Research, University of Split School of Medicine, 21000 Split, Croatia; 4 Clinical Department of Diagnostic and Interventional Radiology, University Hospital of Split, 21000 Split, Croatia; gdujic@kbsplit.hr; 5 Department of Integrative Physiology, University of Split School of Medicine, 21000 Split, Croatia; zeljko.dujic@mefst.hr

**Keywords:** cannabidiol, cardiovascular disease, endocannabinoid system, anti-inflammatory

## Abstract

Cannabidiol (CBD) and other phytocannabinoids are gaining attention for their therapeutic potential in cardiovascular disease (CVD), the world’s leading cause of death. This review highlights advances in understanding the endocannabinoid system, including CB1 and CB2 receptors, and the mechanisms by which CBD exerts anti-inflammatory, antioxidative, vasoprotective, and immunomodulatory effects. Preclinical and translational studies indicate that selective activation of CB2 receptors may attenuate atherogenesis, limit infarct size in ischemia–reperfusion injury, decrease oxidative stress, and lessen chronic inflammation, while avoiding the psychotropic effects linked to CB1. CBD also acts on multiple molecular targets beyond the CB receptors, affecting redox-sensitive transcription factors, vascular tone, immune function, and endothelial integrity. Early clinical trials and observational studies suggest that CBD may lower blood pressure, improve endothelial function, and reduce sympatho-excitatory peptides such as catestatin, with a favorable safety profile. However, limited bioavailability, small sample sizes, short study durations, and uncertainty about long-term safety present challenges to its clinical use. Further research is needed to standardize dosing, refine receptor targeting, and clarify the role of the endocannabinoid system in cardiovascular health. Overall, current evidence supports CBD’s promise as an adjunct in CVD treatment, but broader clinical use requires more rigorous, large-scale studies.

## 1. Introduction

*Cannabis sativa* L., commonly known as hemp, has been cultivated and utilized medicinally across Eurasia with documented mentions in Chinese pharmacopeial texts as early as 2000 BC. Early records, including Papyrus Ramesseum III (~1550 BC), describe the use of *Cannabis* preparations to alleviate ailments such as ocular disorders [1]. Across diverse cultures, from India and Egypt to the Islamic regions, *Cannabis* was used for pain management, antiemetic, anticonvulsant, and anti-inflammatory purposes [2]. In the late 1830s, Western medicine recognized *Cannabis* for its therapeutic potential. William O’Shaughnessy introduced it as a treatment for tetanus and convulsions, while Moreau de Tours explored its psychotropic effects [3]. 

Despite this momentum, the active constituents remained unidentified until scientists from the University of Jerusalem elucidated their stereochemistry in the late 1960s. Delta-9-tetrahydrocannabinol (THC) was first isolated, and at the time, its hydrophobic properties were linked to its non-specific effects [4]. Since then, more than 120 cannabinoids have been discovered, yet only THC and CBD have undergone extensive pharmacological research. THC was established as the principal psychoactive cannabinoid [5]. Still, it was not until the late 1980s and early 1990s that CB1 and CB2 receptors were discovered, followed by the identification of endogenous ligands such as anandamide and 2-arachidonoylglycerol (2-AG). Together with the enzymes responsible for their synthesis and degradation, these components define the endocannabinoid system (ECS), a complex cell-signaling system involved in regulating diverse physiological processes, including pain, mood, appetite, and immune response [5].

Clinical and predominantly preclinical studies have highlighted specific *Cannabis*-derived compounds, THC, CBD, and their combination nabiximols, as having established clinical indications for conditions such as chronic pain, spasticity, cachexia, and certain forms of epilepsy (for CBD). Additionally, off-label use and preclinical evidence suggest potential therapeutic roles for these compounds in managing anxiety, nausea, and post-traumatic stress disorder [6]. Moreover, the aforementioned elucidation of endocannabinoid signaling mechanisms has reshaped the discourse around *Cannabis*, from mystical associations and legal prohibitions to evidence-based therapeutic applications grounded in molecular pharmacology [7,8]. Nowadays, cannabidiol has garnered attention for its vasodilatory, anti-inflammatory, and antioxidant effects on cardiovascular health. With this growing body of evidence and focused scientific interest, it is essential to contextualize these findings within the broader landscape of cardiovascular disease (CVD) [9].

Globally, CVD remains the leading cause of mortality, accounting for approximately 19.8 million deaths in 2022, which represents over a quarter of all global deaths and an increase from 12.4 million deaths in 1990, reflecting global population growth and aging, as well as the impact of modifiable risk factors [10]. In 2021, more than 612 million people were diagnosed with some form of CVD, with the vast majority of morbidity and mortality (around 80%) occurring in low and middle-income countries. Although age-standardized mortality rates have declined in recent decades due to improved prevention and treatment strategies, the absolute number of deaths continues to rise, driven by population growth and aging. In Europe alone, CVDs are responsible for approximately 42.5% of all-cause mortality, with disproportionately high rates observed in Eastern Europe [11]. These data underscore the urgent need for innovative and accessible therapeutic strategies, such as cannabidiol, to complement existing cardiovascular prevention and treatment efforts, particularly in resource-limited settings.

## 2. Cannabidiol, Cannabinoid Receptors, and Endocannabinoids

### 2.1. Cannabinoid Receptors: Structure, Distribution, and Signaling Pathways

The specific cellular signaling mechanism via two membrane-bound receptors was demonstrated in the early 1990s. First, the cannabinoid receptor type 1 (CB1) was independently cloned from rat and human brain cDNA libraries [12,13]. CB1 is a G-protein-coupled receptor that modulates cellular activity through several pathways. First, it suppresses adenylate cyclase activity, leading to decreased levels of cAMP. Second, it inactivates A-type K+ channels and inhibits neurotransmitter release by enabling the β/γ subunits of the associated G protein to directly bind with and inhibit voltage-gated calcium channels in the postsynaptic membrane [14]. It is most prominently expressed by central neurons, especially in the cerebral cortex and hippocampus [15]. In addition to the remaining levels of the autonomic nervous system and the peripheral nervous system, it is necessary to highlight the expression of CB1 in the circulatory system, particularly in the heart [16]. However, attempts to target CB1 therapeutically have been limited by unwanted psychotropic side effects despite its role in the regulation of energy metabolism and lipogenesis. Namely, to date, targeted CB-1 therapy has only been approved once in 2006. The CB1 antagonist, rimonabant, was used for treating obesity. Only two years later, despite achieved weight loss, increased HDL, and decreased plasma triglycerides and HbA1c, its production was discontinued due to reports of depression and suicidality, most likely caused by effects on CB1 in the CNS [17,18,19].

Shortly thereafter, CB2 was discovered and cloned from HL60 cells (a human promyelocytic leukemia line). As with CB1, the CB2 receptor is also a member of the G protein-coupled receptor (GPCR) superfamily, characterized by seven transmembrane α-helices interconnected by three extracellular and three intracellular loops, with an N-terminal glycosylated extracellular domain and a cytoplasmic C-terminus [20]. While CB1 and CB2 are encoded by distinct intronless genes (*CNR1* and *CNR2*, respectively), their structural features reflect significant evolutionary conservation. The *CNR1* gene is located on human chromosome 6, whereas *CNR2*, is located on the first chromosome. Despite functional divergence, CB1 and CB2 share approximately 45% overall amino acid sequence identity, with notably higher homology (around 70%) confined to the transmembrane domains, critical components for ligand binding and signal transduction [21]. Furthermore, CB2 demonstrates considerable interspecies conservation. For instance, the human and rodent CB2 receptor sequences exhibit over 80% identity, underscoring their preserved physiological relevance across mammalian systems. Although CB1 expression has also been reported in immune cells, its levels are significantly lower levels than those of CB2 [22]. 

CB-2 is primarily expressed on peripheral immune cells, especially those activated in the inflammatory cascade, such as interferon-γ-stimulated macrophages [23]. Its expression in other, or “non-immune cells”, is less pronounced. It has been observed in only a few cell types, including cardiomyocytes, endothelial cells, and vascular smooth muscle cells [24,25,26,27].

CB2 receptors are G protein-coupled receptors (GPCRs) that predominantly act through Gi/o proteins, which are sensitive to pertussis toxin. This signaling cascade inhibits adenylate cyclase activity, resulting in reduced intracellular levels of cyclic adenosine monophosphate (cAMP) and subsequent downregulation of protein kinase A (PKA) activity. The CB2 receptor further modulates several intracellular signaling cascades across various cell types, including the phospholipase C pathway, the Janus kinase/signal transducer and activator of transcription (JAK/STAT) pathway, and the nuclear factor kappa-light-chain-enhancer of activated B cells (NF-κB) pathway. These signaling pathways allow CB2-mediated modulation of critical cellular functions, including proliferation, migration, and survival of immune cells. This results in immunosuppression that may have therapeutic relevance [28].

### 2.2. Pharmacodynamics of Cannabidiol

The distinct physiological effects of phytocannabinoids, particularly THC and CBD, are primarily attributed to their differential affinities for both cannabinoid and non-cannabinoid receptors, resulting in modulation of the ECS [29]. THC exhibits a high binding affinity for cannabinoid receptors. Activation of CB1 by THC is responsible for its psychoactive effects, manifesting as alterations in perception, mood, and anxiety, whereas CB2 activation is implicated in the regulation of inflammatory processes and immune function [30].

**Figure 1:**
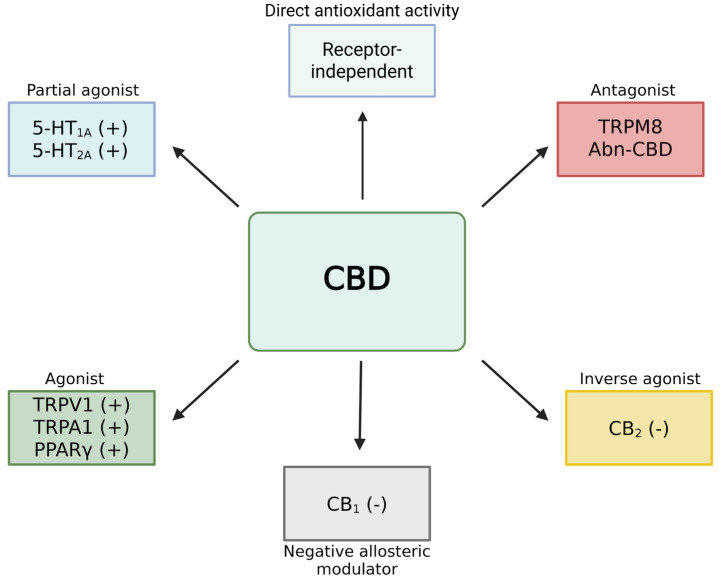
Pleiotropic effects of cannabidiol (CBD). CBD acts through multiple mechanisms, including receptor-dependent modulation as a partial agonist (5-HT1A, 5-HT2A), agonist (TRPV1, TRPA1, PPARγ), antagonist (TRPM8, Abn-CBD), inverse agonist (CB2), and negative allosteric modulator (CB1). Importantly, CBD also exerts a direct receptor-independent antioxidant effect by scavenging free radicals and modulating endogenous antioxidant systems. Abbreviations: CB1: cannabinoid receptor type 1; CB2: cannabinoid receptor type 2; 5-HT: serotonin receptor; Abn-CBD: abnormal cannabidiol; PPARγ: peroxisome proliferator-activated receptor gamma; TRPV1: vanilloid receptor type 1; TRPA1: ankyrin-type receptor A1 from the TRP ion channel family; TRPM8: melastatin-type receptor M8 from the TRP ion channel family.

In contrast, CBD displays a relatively low binding affinity for both CB1 and CB2 receptors. It functions as a negative allosteric modulator at the CB1 receptor, thereby attenuating the psychoactive effects of THC. Additionally, CBD exerts partial agonist or modulatory effects on various non-cannabinoid receptors, including serotonergic receptors (5-HT1A and 5-HT2A), transient receptor potential vanilloid type 1 (TRPV1), transient receptor potential ankyrin 1 (TRPA1), and the nuclear peroxisome proliferator-activated receptor gamma (PPARγ) (Figure 1). These receptor interactions contribute to CBD’s broad pharmacological profile [31] (Figure 1). Furthermore, CBD has been identified as a positive allosteric modulator of several glycine receptor subtypes (α1, α1β, and α3), μ- and δ-opioid receptors, and gamma-aminobutyric acid type A (GABA-A) receptors. In contrast, it negatively modulates α1-adrenergic, dopamine D2, and serotonin 5-HT3 receptors. This receptor diversity underlies CBD’s multifaceted pharmacological profile, which encompasses anxiolytic, anticonvulsant, anti-inflammatory, and neuroprotective effects [32]. CBD has also demonstrated immunosuppressive properties. Experimental studies have demonstrated that inhibition or antagonism of CB1, CB2, and TRPV1 receptors abolishes THC-induced immunomodulatory effects. Additionally, CBD inhibits key signaling pathways, including Janus kinase/signal transducer and activator of transcription (JAK/STAT) and the nucleotide-binding oligomerization domain-like receptor (NLR) pathways, resulting in decreased synthesis and release of pro-inflammatory cytokines. Moreover, CBD modulates adenosine receptors indirectly by inhibiting the equilibrative nucleoside transporter ENT1, leading to increased extracellular adenosine levels and enhanced receptor activation. This mechanism is a key contributor to CBD’s cytoprotective and anti-inflammatory properties. Thus, modulation of adenosine receptors represents another significant receptor-mediated pathway underlying CBD’s therapeutic effects, complementing its actions on serotonergic, TRP, and nuclear receptors [31,33].

The defining feature of phytocannabinoids is their capacity to interact with the ECS, a complex network comprising endocannabinoids (e.g., anandamide, 2-arachidonoylglycerol), their receptors, uptake transporters, and associated metabolic enzymes responsible for synthesis and degradation. The ECS plays a crucial role in maintaining systemic homeostasis, influencing diverse physiological processes, including nociception, appetite, immune responses, emotional regulation, and cardiovascular function [34]. Phytocannabinoids modulate the ECS via distinct mechanisms. THC acts as a partial agonist at CB1 and CB2 receptors, while CBD inhibits the enzymatic degradation of endocannabinoids, thereby increasing endogenous levels of anandamide and 2-AG. This elevation may lead to physiological effects such as peripheral vasodilation or compensatory tachycardia [35].

The interaction between THC and CBD is characterized by complexity and dose-dependency. For instance, low doses of CBD may mitigate THC-induced anxiety, whereas higher doses may enhance its sedative properties. This bidirectional modulation necessitates careful consideration when co-administering phytocannabinoids, as the therapeutic or adverse outcomes may vary significantly depending on the ratio and dose administered [36].

Importantly, while CBD demonstrates a wide range of biological activities through interactions with multiple receptor systems, much of the supporting evidence is derived from in vitro or animal model studies. Consequently, caution is warranted when extrapolating these findings directly to clinical settings, and further translational research is essential to substantiate the therapeutic efficacy of CBD across various pathologies [37].

### 2.3. The Endocannabinoid System

The prior discovery and molecular characterization of cannabinoid receptors provided a foundation for identifying their endogenous ligands (endocannabinoids) as well as the enzymes responsible for their synthesis and degradation. The key endocannabinoids include anandamide (AEA; N-arachidonoylethanolamide), 2-arachidonoylglycerol (2-AG), N-arachidonoyldopamine (NADA), and virodhamine (O-arachidonoylethanolamine) [38]. Together with the CB1 and CB2 receptors, uptake mechanisms, and associated metabolic enzymes, these lipid-derived signaling molecules constitute the ECS, a complex and highly conserved neuromodulatory network involved in regulating a wide array of physiological processes [39].

Endocannabinoids are bioactive lipid mediators with a common basic form derived from their membrane phospholipid precursors, which are composed of arachidonic acid. The stimulation of neurons, platelets, and activated macrophages most often elicits them. The first two listed endocannabinoids are produced and further released when the intracellular calcium concentration increases [40]. The synthesis of 2-AG consists of two simple steps, where sn-1-acyl-2-arachidonoylglycerols (DAG) are first generated by phospholipase C-mediated hydrolysis of phosphatidyl-inositols and then converted to 2-AG by the action of one of two Ca^2+^-sensitive sn-2-selective DAG lipases (DAGLα and DAGLβ) [41]. 

Powell and others have proven that knockout of the DAGLα gene produces similar behavioral phenotypes and body weight compared to those of CB1 receptor knockout mice, further underscoring the pivotal role of 2-AG and CB1 within the central nervous system in regulating feeding behavior, maintaining energy balance, and promoting metabolic homeostasis [42]. The synthesis of AEA is somewhat more complicated and follows the same two-part process as 2-AG synthesis. Firstly, by the action of a Ca2+-dependent N-acyltransferase, an acyl chain is transferred to phosphatidylethanolamine (PE) to form N-arachidonoyl phosphatidylethanolamine (NAPE). EA (anandamide, also known as N-arachidonoylethanolamine) is then synthesized from NAPE through the hydrolysis catalyzed by a calcium-sensitive, NAPE-specific phospholipase D (NAPE-PLD). However, studies in NAPE-PLD knockout mice suggest the presence of alternative biosynthetic pathways, indicating that multiple NAPE-PLD-independent pathways can contribute to AEA production [43]. 

Understanding of the dynamic alterations in the ECS during damage to the myocardium represents a crucial point to establish novel cannabinoid-based therapeutic approaches. Preclinical and clinical data confirm ECS is involved in diverse physiological and pathological processes. Alterations in receptor expression and endocannabinoid levels have been associated with neurological conditions, metabolic disorders, cardiovascular diseases, and certain cancers [44]. For example, in a state of imbalance in the ECS and increased expression of CB1, the action of anandamide and further activation of downstream pathways result in increased oxidative stress and apoptotic signaling [45].

## 3. CB2 Receptor Activation and Anti-Inflammatory Mechanisms in Cardiovascular Diseases

CB2 has emerged as a modulator of cardiovascular health, exerting potent anti-inflammatory and atheroprotective effects while avoiding the psychotropic activity associated with CB1 activation [46]. Mechanistically, CB2 counteracts pro-atherogenic processes by modulating key signaling pathways. In human monocytes, CB2 agonists downregulate chemokine receptors (CCR1, CCR2) and adhesion molecules, thereby reducing chemotaxis toward CCL2 and CCL3. Furthermore, CB2 activation promotes the polarization of anti-inflammatory macrophages, downregulates NF-κB activity, and reduces the secretion of pro-inflammatory cytokines in foam cells. In vascular smooth muscle cells, CB2 stimulation mitigates TNF-α-induced proliferation and migration via MAPK/ERK and PI3K/Akt signaling [47].

Preclinical studies in ApoE^−/−^ mice have confirmed that CB2-selective agonists such as WIN55,212-2 and JWH-133 reduce atherosclerotic burden by inhibiting endothelial activation, decreasing adhesion molecule expression (VCAM-1, ICAM-1, P-selectin), and suppressing macrophage infiltration [48]. In myocardial infarction models, administration of 2-AG and palmitoylethanolamide before ischemia demonstrated cardioprotective effects, notably reducing infarct size and cellular damage. These effects are abolished by CB2 antagonists, underscoring receptor specificity [49].

CB2 activation also exerts anti-arrhythmic effects, likely via inhibition of cAMP accumulation, which suppresses calcium overload and electrical instability. Additional studies have demonstrated that CB2 modulates transient receptor potential (TRP) channels, which are involved in maintaining electrophysiological stability, thereby reducing arrhythmogenic potential [50].

Genetic deletion of CB2 in murine models exacerbates vascular oxidative stress and accelerates the development of atherosclerotic lesions, underscoring its protective function. In balloon injury models, CB2 agonism inhibits neointimal hyperplasia by restraining smooth muscle proliferation and inflammatory cell recruitment [51].

In ischemia–reperfusion injury, stimulation of the CB2 receptor attenuates leukocyte recruitment, reduces infarct size, and enhances survival pathways in cardiomyocytes and endothelial cells. These effects occur without central nervous system side effects, making CB2 a promising therapeutic target [52].

Evidence also supports the activation of CB2 in heart failure models. Agonists enhance diastolic function, mitigate ventricular remodeling, and suppress the expression of genes associated with fibrosis. Such effects are partly mediated through suppression of ROS generation, upregulation of nuclear factor erythroid 2-related factor 2 (Nrf2), and inhibition of caspase activation, collectively contributing to cardiomyocyte survival and mitochondrial stability [53]. 

**Figure 2:**
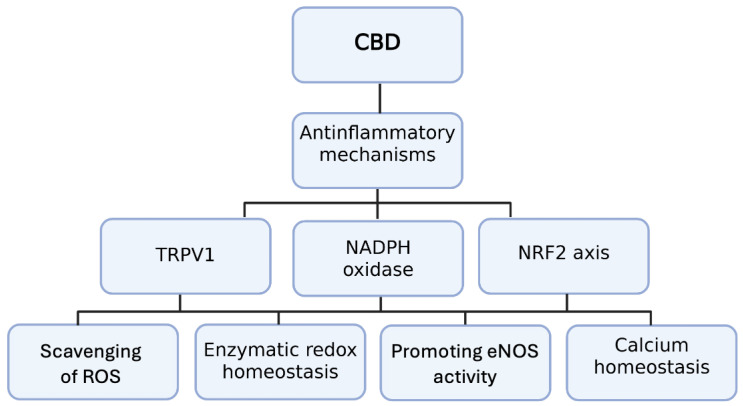
Anti-inflammatory mechanisms of cannabidiol (CBD) involving TRPV1, NADPH oxidase, and the NRF2 axis. Abbreviations: CBD: cannabidiol; CB1: cannabinoid receptor type 1; eNOS: endothelial nitric oxide synthetase; TRPV1: vanilloid receptor type 1; NADPH: nicotinamide adenine dinucleotide phosphate; NRF-2: Nuclear factor erythroid–2-related factor 2.

Of important note, cross-talk between CB1 and CB2 receptors in inflammatory signaling may influence therapeutic outcomes. While CB1 activation generally promotes oxidative stress and inflammation, CB2 stimulation attenuates these effects, highlighting the need to selectively modulate cannabinoid receptor pathways in cardiovascular settings [54]. However, it should be noted that many of the described vasorelaxant and cardioprotective effects have been demonstrated only in preclinical models, often in rodents exposed to supraphysiological doses of CBD, thus limiting our ability to adjudicate cardiovascular effects of these molecules. Antioxidant and anti-inflammatory pathways od CBD are summarized in Figure 2.

## 4. Molecular Effects of CBD on Cardiovascular Pathophysiology

The cardiovascular effects of CBD have become a focal point in recent research, particularly in the context of its impact on inflammatory cascades, which play an essential role in the development of cardiovascular diseases [55]. CBD exerts complex and pleiotropic actions through interactions with a range of molecular targets, including ECS receptors, non-cannabinoid receptors, and redox-sensitive transcription factors. In particular, increasing evidence indicates the beneficial effects of CBD on the cardiovascular system. Those effects include suppression of pro-inflammatory cytokine release, inhibition of macrophage activation, and attenuation of oxidative stress [56]. Such mechanisms contribute to the potential reduction in risk and progression of conditions such as atherosclerosis, arterial hypertension, and heart failure [57]. Although exogenous CBD may not precisely replicate the effects of endogenous cannabinoids, its regulatory influence on the cardiovascular system is evident. For instance, levels of anandamide, a key ECS ligand with vasoregulatory properties, are often altered in hypertensive and diabetic patients [58]. CBD may improve endothelial function, reduce heart rate, and exert cardioprotective effects during stress exposure (possibly associated with anxiolytic effects), partially through modulation of autonomic tone and reduction in sympathetic drive [59].

Chronic low-grade inflammation is now recognized as a fundamental feature in the pathogenesis of cardiovascular disorders [60]. Pro-inflammatory mediators, such as IL-1β, IL-6, IL-8, IL-18, and TNF-α, contribute to endothelial dysfunction and increased systemic vascular resistance (SVR), ultimately leading to elevated arterial pressure. The lectin-like oxidized LDL receptor further amplifies vascular injury by promoting endothelial activation, platelet aggregation, and apoptosis, three key cornerstones in atherogenesis [61]. Furthermore, plasminogen activator inhibitor-1, known for its pronounced prothrombogenic effect, is also involved in atherosclerosis development. Thus, the pathophysiology of cardiovascular diseases is intricately tied to immune system overactivation, driven by endogenous factors such as oxidative stress and exogenous factors like obesity and tobacco consumption [62]. CBD’s immunomodulatory capacity may counteract these pathological stimuli, thereby offering mechanistically grounded therapeutic potential.

Among enzymatic sources of oxidative damage, NADPH oxidase plays a pivotal role in generating reactive oxygen species (ROS) in the cardiovascular system. In addition, degradation of nitric oxide synthase in oxidative environments shifts the role of nitric oxide from vasoprotection to facilitating superoxide production, thereby worsening vascular injury [63]. Endothelial dysfunction subsequently promotes vasospasm, thrombosis, and tunica media proliferation, perpetuating vascular inflammation [64].

**Figure 3:**
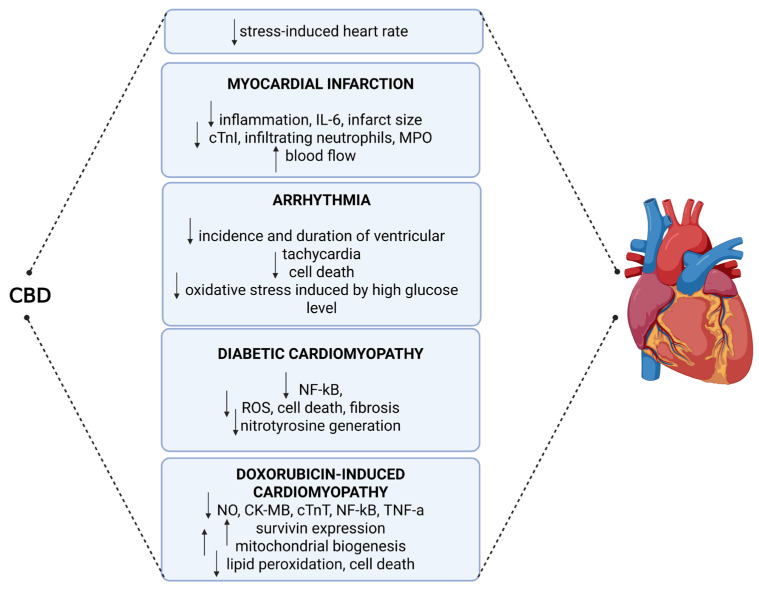
Cardioprotective Effects of Cannabidiol (CBD) on Cardiovascular Disorders. CBD: cannabidiol; IL-6: interleukin-6; cTnI: cardiac troponin I; MPO: myeloperoxidase; NF-κB: nuclear factor kappa-light-chain-enhancer of activated B cells; ROS: reactive oxygen species; NO: nitric oxide; CK-MB: creatine kinase-MB; cTnT: cardiac troponin T; TNF-α: tumor necrosis factor-alpha.

CBD demonstrates both direct and indirect antioxidant activity. Its phenolic structure enables scavenging of reactive species, while coordination with transition metal ions attenuates Fenton reaction-driven radical formation [65]. CBD upregulates antioxidant defenses *via* the Nrf2/Keap1 pathway, increasing transcription of enzymes such as superoxide dismutase (SOD) and glutathione peroxidase. Additionally, CBD prevents the depletion of essential trace elements, such as selenium and zinc, which are crucial for maintaining enzymatic redox homeostasis. Through its inverse agonistic effects on CB2 receptors, CBD reduces circulating TNF-α levels and ROS production, distinguishing its effects from those of CB1 receptor stimulation, which generally exacerbates inflammation [66]. CBD’s interaction with TRPV1 modulates calcium homeostasis, another critical component of inflammatory signaling [67]. Furthermore, its action on PPARγ not only inhibits NF-κB and COX-2 transcription but also induces genes encoding catalase, Mn-SOD, and heme oxygenase-1, reinforcing cytoprotection via the Nrf2 axis. CBD also inhibits degradation of anandamide and 2-AG, further potentiating PPARγ activation [66,68]. By engaging 5-HT1A receptors, CBD limits oxidative damage induced by lipid peroxidation. Moreover, its immunosuppressive effects include regulation of IL-1β and IL-6 expression in monocytes activated via Toll-like receptors [69]. Notably, Zaiachuk et al. demonstrated that IL-6 and IL-10 are exceptionally responsive to CBD pre-treatment in human macrophages. In the context of cardiovascular pathology, CBD’s anti-inflammatory and antioxidant properties may underlie its hypotensive effects. By suppressing inflammatory cytokines, reducing oxidative stress, and downregulating RAAS activity, CBD lowers SVR and ameliorates endothelial function [70]. These pleiotropic mechanisms and their cardioprotective consequences are illustrated in Figure 3.

In summary, low-grade systemic inflammation acts as a central mechanism in the pathophysiology of cardiovascular diseases. Through modulation of oxidative and inflammatory pathways, CBD presents a promising agent for antihypertensive and vasoprotective effects, deserving further investigation.

## 5. Translational Evidence and Therapeutic Outlook: Clinical Findings and Experimental Insights

CBD’s complex and pleiotropic pharmacodynamics have sparked extensive interest in its cardioprotective potential. Despite promising preclinical data, the cardiovascular effects of this compound remain incompletely defined in humans [71].

In animal and in vitro models, it consistently demonstrates vasodilatory properties and improvement of endothelial function. However, studies in hypertensive rodent models suggest that baseline resting blood pressure often remains unaffected by chronic treatment [72]. Remiszewski et al. reported no significant decrease in blood pressure in rats with either primary or secondary hypertension after long-term CBD exposure, despite observable improvements in lipid metabolism and oxidative stress [73].

In contrast, under acute or stress-induced conditions, CBD exerts clear antihypertensive effects. A meta-analysis by Sultan et al. confirmed reductions in blood pressure responses during stress, likely linked to anxiolytic and autonomic mechanisms [74].

Flôr et al. documented that chronic CBD administration in renin-responsive hypertensive rats lowered arterial pressure, improved baroreflex sensitivity, and reduced vascular oxidative stress [75]. Similarly, Baranowska-Kuczko et al. reported a notable decrease in systolic blood pressure and heart rate in hypertensive rats, along with enhanced endothelium-dependent vasodilation [76]. In acute ischemia–reperfusion models, Walsh et al. provided evidence of reduced infarct size and arrhythmogenesis following intravenous pre-treatment [77]. Moreover, Durst et al. documented a marked ~66% decrease in infarct size and reduced myocardial inflammation in rat MI models while Franco-Vadillo and his group underscored enhancements in cardiomyocyte survival *via* PI3K/Akt and MAPK/ERK signaling [78,79,80].

These findings collectively support the antioxidative, anti-inflammatory, and anti-apoptotic capacities of cannabinoids. The central role of CB2, which lacks the psychoactive effects of CB1, provides a safer therapeutic window. Inhibition of MAPK and PI3K/Akt signaling also contributes to vascular homeostasis. Overall, these studies advocate further exploration of cannabinoids, particularly CB2 agonists as adjunctive therapies in CVD.

On the other hand, preliminary clinical studies exploring CBD in the context of cardiovascular conditions have yielded promising outcomes. Jadoon et al. observed a reduction in resting blood pressure and stress responses in healthy subjects after a single oral dose of CBD [81]. Similarly, Sultan and colleagues described the reduction in arterial stiffness and improvements in endothelial function after repeated CBD dosing in response to isometric exercise [82]. To expand upon these observations, several recent clinical studies, particularly from Central European cohorts, have provided novel insights into the cardiovascular effects of chronic CBD administration. Our research group has conducted a series of studies investigating the cardiovascular effects of CBD formulations. Initially, we examined the effects of a single dose of TurboCBD™ (Lexaria Bioscience Corp., Kelowna, BC, Canada), a patented formulation in which long-chain fatty acids rich in oleic acid are conjugated with CBD through a dehydration-based process [83]. This approach enhances CBD uptake by reducing first-pass metabolism. That study demonstrated higher systemic CBD concentrations compared with a standard formulation, along with increased cerebral perfusion and a signal for BP–lowering effect. Subsequently, we investigated an optimized version of the formulation (DehydraTECH™2.0, Lexaria Bioscience Corp., Kelowna, BC, Canada; CBD; 150 mg every 8 h for 24 h) in a crossover pilot study in hypertensive individuals [84]. Building on these results, we conducted a double-blind, placebo-controlled, crossover trial (HYPER-H21-4), which aimed to determine the BP–lowering effects of chronic CBD administration (5 weeks) in patients with hypertension. This trial showed that chronic oral CBD significantly reduced 24-h ambulatory blood pressure without affecting arterial stiffness in patients with primary hypertension. Importantly, these effects occurred without clinically relevant adverse events or changes in liver enzymes, supporting both the safety and cardiovascular potential of CBD [85]. In addition, we performed several substudies to explore the mechanisms underlying CBD-mediated blood pressure reduction. Chronic CBD administration significantly lowered serum catestatin, a sympathoinhibitory peptide implicated in hypertension pathophysiology. This reduction correlated with decreases in mean arterial pressure, suggesting modulation of the sympatho-chromaffin axis [86]. Similarly, five weeks of CBD supplementation significantly reduced serum urotensin, a potent vasoconstrictor and marker of vascular stress, with the magnitude of urotensin reduction positively correlating with decreases in 24-h mean arterial pressure [87]. Furthermore, CBD administration increased circulating AEA but not 2-AG levels, although these changes were not associated with the observed blood pressure reduction, providing no conclusive evidence that endocannabinoid modulation accounts for the effect (study currently under revision). Pharmacogenetic influences were also explored, as detailed in the study by Batinic et al. [88]. Beyond vascular outcomes, CBD supplementation was associated with improvements in fatigue and daytime sleepiness, suggesting additional quality-of-life benefits [89]. 

Together, these findings highlight potential benefits across vascular, metabolic and sympathetic domains. Nevertheless, the relatively small sample sizes call for larger, well-designed randomized controlled trials. Future clinical studies should also address long-term outcomes and optimal dosing strategies positioning CBD as a potential adjunct in cardiovascular care. 

Table 1 and Table 2 provide an overview of preclinical and early clinical findings on the use of cannabinoids in cardiovascular disease.

From a safety perspective, CBD demonstrates a favorable profile. Unlike THC, it does not cause reflex tachycardia or acute coronary events [90,91]. Meta-analyses indicate that chronic CBD dosing is associated with mild to moderate adverse effects, primarily gastrointestinal, such as diarrhea or nausea, and CNS-related, including somnolence and headache. Notably, no significant cardiovascular toxicity has been reported [92,93]. However, high-dose CBD has exhibited cardiovascular toxicity and hepatotoxicity in preclinical settings, alongside endocrine disruption and reduced fertility [94,95,96]. Drug interactions, particularly with agents like bupropion, propofol, and lorazepam, must be considered [97]. Further supporting the complexity of CBD pharmacology, Batinic et al. identified sex-dependent differences in plasma CBD concentrations, highlighting the importance of personalized dosing strategies [98].

Nonetheless, most human studies are limited in size and focus on short-term or acute dosing. The lack of robust, long-term randomized trials represents a significant translational gap [99]. Additionally, interindividual variability in ECS activity and CBD metabolism further complicates the standardization of therapy [100]. Future therapeutic directions may include the development of CB2-selective agonists, FAAH inhibitors, and peripheral CB1 antagonists to avoid central side effects. Strategies such as nanoparticle delivery systems and dual-targeting compounds may enhance bioavailability and precision [101].

On the other hand, despite growing interest, human clinical data remain limited and heterogeneous. Most trials involve acute dosing in healthy volunteers, not patients with cardiovascular pathology, and report modest haemodynamic changes, such as transient reductions in blood pressure, not necessarily replicable at chronic, therapeutic dosing.

In conclusion, while preclinical and limited clinical data offer compelling evidence for CBD’s cardioprotective role, clinical application requires further exploration to resolve dosing, safety, and efficacy parameters [102].

## 6. Challenges, Knowledge Gaps, and Future Directions

Despite the growing body of preclinical and clinical research into the cardiovascular effects of CBD and other cannabinoids, several limitations persist that hinder their full integration into cardiovascular therapeutics. One of the most pressing issues is the translational gap between experimental findings and clinical application [103] (Figure 3). Although preclinical studies consistently demonstrate anti-inflammatory, antioxidative, vasorelaxant and cardioprotective properties, primarily mediated through CB2 receptor activation, PPARγ engagement and modulation of the Nrf2 signaling axis, clinical investigations remain limited in number and scope, often constrained by small sample sizes, short intervention durations and the inclusion of predominantly healthy individuals or narrowly defined patient cohorts [104]. Key knowledge gaps include the long-term cardiovascular safety of chronic CBD use, the variability in efficacy due to interindividual differences in ECS activity, and the lack of standardized dosing protocols. Furthermore, most currently available cannabinoid formulations suffer from poor oral bioavailability and limited peripheral selectivity, raising concerns about systemic distribution and central nervous system side effects, particularly with CB1 modulation [105,106].

Another challenge lies in the complex pharmacodynamics of CBD, which acts on multiple receptor systems, and its effects can vary depending on the specific biological context or environment [107]. This pleiotropy complicates the interpretation of results and highlights the need for targeted delivery systems, such as nanoparticle-based carriers or peripheral CB1 antagonists, to optimize therapeutic precision while minimizing adverse effects. Additionally, the potential for drug–drug interactions, especially with commonly prescribed cardiovascular and neuroactive medications, must be systematically evaluated [108].

Nevertheless, the therapeutic promise of cannabinoids remains substantial. CBD’s ability to modulate vascular tone, attenuate systemic inflammation, and enhance endothelial function, combined with a generally favorable safety profile and lack of psychotropic effects, positions it as a compelling candidate for adjunctive cardiovascular therapy [109]. However, the clinical applicability of cannabidiol across all the aforementioned domains remains incompletely clarified. Notably, much of the supporting data is from preclinical studies, and robust human evidence, especially from large, long-term, placebo-controlled trials, is still lacking and significantly tempering enthusiasm for clinical translation [110].

**Figure 4:**
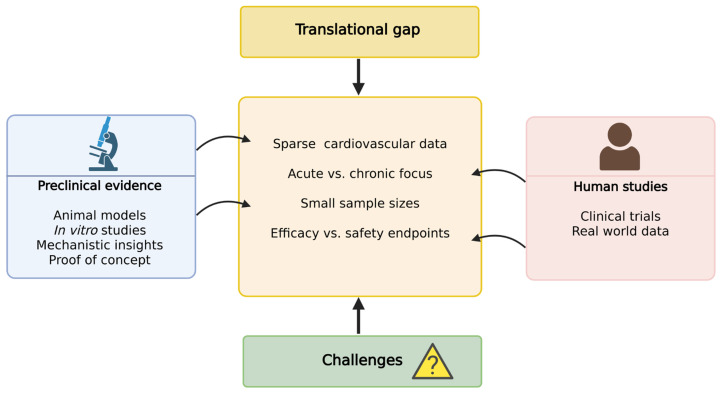
Translational gap between preclinical findings and human studies on the cardiovascular effects of cannabidiol (CBD).

Future directions should focus on large-scale, placebo-controlled, randomized clinical trials, the development of ECS-targeting compounds with improved pharmacokinetics, and elucidation of the mechanisms underlying ECS signaling in cardiovascular physiology and pathology [111]. Research priorities should also address long-term safety and tolerability, rigorous pharmacokinetic and pharmacodynamic profiling across diverse populations, and innovative strategies to enhance bioavailability, such as lipid-based formulations, nanocarriers, or prodrugs, to improve clinical translation [112]. Well-powered trials are further needed to establish efficacy, define optimal dosing, and clarify the role of ECS signaling in vascular regulation and systemic inflammation in both healthy individuals and patients with cardiovascular disease [113]. Finally, a comprehensive evaluation of drug–drug interactions, particularly with widely used cardiovascular medications, will be critical to ensure safety in clinical practice. Figure 4 summarizes the translational gap between preclinical evidence and human studies, outlining the key challenges that hinder effective translation of findings.

In summary, although cannabinoids have not yet reached clinical maturity for broad implementation in cardiovascular therapeutics, the underlying scientific evidence is promising, and preliminary findings are encouraging. Bridging the translational gap prudently will demand methodologically rigorous, multidisciplinary research efforts, ensuring that any future clinical claims are grounded in robust human evidence.

## Figures and Tables

**Table 1:** Overview of preclinical animal studies demonstrating the cardiovascular, vasoprotective, and anti-inflammatory effects of cannabidiol (CBD) in various disease models.

Study	Animal Model	Disease	Findings
[75]	Male Wistar rats	Renovascular hypertension	CBD loweredarterial pressure, improved baroreflex sensitivity, and reduced vascular oxidative stress
[76]	Wistar–Kyoto rats	Primary and secondary hypertension	CBD vasoprotective effects in hypertensive rats, via inducing local vascular changes in the ECS
[77]	Male Sprague-Dawley rats	Acute ischemia–reperfusion model	CBD reduced ventricular arrhythmias and attenuated infarct size
[78]	Ligating-induced MI in rats	Heart failure after MI	CBD improved cardiac function and reduced infarct size via anti-inflammatory pathways
[80]	Male Wistar rats	Monocrotaline- induced PAH	CBD improved endothelial efficiency and function, reduced RVSP and pulmonary vascular remodeling, normalized hemostatic alterations

Abbreviations: CBD: cannabidiol; MI: myocardial infarction; ECS: endocannabinoid system; PAH: pulmonary arterial hypertension.

**Table 2:** Summary of clinical studies evaluating the cardiovascular effects of cannabidiol (CBD) in human populations.

Study	Population (N)	Condition	CBD Dosage	Findings
[81]	9 healthy male volunteers	Without documented diseases	600 mg orally (single dose)	CBD reduced resting systolic blood pressure and attenuated blood pressure response to stress
[82]	10 healthy volunteers	Stress-related cardiovascular response	600 mg orally for seven days	CBD reduced arterial stiffness and improved endothelial function after repeated dosing in response to stress
[84]	16 patients with untreated Grade 1 and Grade 2 hypertension	Primary hypertension	150 mg every 8 h orally	CBD lowers systolic and mean BP and arterial stiffness
[85]	64 patients with mild or moderate hypertension, untreated or receiving standard of care therapy	Primary hypertension	CBD orally (225–450 mg) for 5 weeks	CBD reduced ambulatory BP and improved daytime alertness

Abbreviations: CBD: cannabidiol; BP: blood pressure.

## Data Availability

No new data were created or analyzed in this study. Data sharing is not applicable to this article.
